# Deconstructing TikTok Videos on Mental Health: Cross-sectional, Descriptive Content Analysis

**DOI:** 10.2196/38340

**Published:** 2022-05-19

**Authors:** Corey H Basch, Lorie Donelle, Joseph Fera, Christie Jaime

**Affiliations:** 1 Department of Public Health William Paterson University Wayne, NJ United States; 2 Arthur Labatt Family School of Nursing Western University London, ON Canada; 3 Department of Mathematics Lehman College City University of New York Bronx, NY United States

**Keywords:** TikTok, mental health, adolescent, social media, short video apps, content analysis, digital health, online health, visual media, descriptive content analysis, mental distress, health professional, health care professional

## Abstract

**Background:**

Social media platforms that are based on the creation of visual media, such as TikTok, are increasingly popular with adolescents. Online social media networks provide valuable opportunities to connect with each other to share experiences and strategies for health and wellness.

**Objective:**

The aim of this study was to describe the content of the hashtag #mentalhealth on TikTok.

**Methods:**

This cross-sectional, descriptive content analysis study included 100 videos with the hashtag #mentalhealth on TikTok. All videos that included the hashtag #mentalhealth were analyzed and coded for the presence of content categories. Additionally, the comments to each video were viewed and coded for content in the following themes: offering support or validation; mentioning experience with suicide or suicidal ideation; mentioning experience with self-harm; describing an experience with hospitalization for mental health issues; describing other mental health issues; and sharing coping strategies, experiences of healing, or ways to feel better.

**Results:**

Collectively, the 100 videos studied received 1,354,100,000 views; 266,900,000 likes; and 2,515,954 comments. On average, each video received 13,406,930.69 (SD 8,728,095.52) views; 2,657,425.74 (SD 1,449,920.45) likes; and 24,910.44 (SD 21,035.06) comments. The only content category observed in most (51/100, 51%) of the videos included in the sample was “general mental health.” The remaining content categories appeared in less than 50% of the sample. In total, 32% (32/100) of the videos sampled received more than the overall average number of likes (ie, more that 2.67 million likes). Among these 32 videos, 23 (72%) included comments offering support or validation and 20 (62%) included comments that described other mental health issues or struggles.

**Conclusions:**

With over 1 billion cumulative views, almost half of the assessed TikTok videos included in this study reported or expressed symptoms of mental distress. Future research should focus on the potential role of intervention by health care professionals on social media.

## Introduction

Access to accurate and accessible health information, support, and services are required for effective health care decision-making. This applies across the health care continuum and is inclusive of self-health promotion and participation in shared decision-making with health care provider teams [1-6]. Individuals rely on family, friends, and health care professionals for support, yet seek health-related information online at the same time [7,8]. In general, adolescents live in a hybrid reality—a mix of offline and online worlds [9]. They are increasingly seeking information about healthy lifestyles (fitness, diet), gender identity, sexual health, and mental health issues from online websites, social media platforms, wearable self-tracking apps, and other adolescents within online communities [10,11]. However, adolescents are less accepting of online or wearable apps that require them to input information [7]. Overall, online media and social media apps are seen as simple to use, unidentifiable, and impartial sources of health information by adolescents [7,12]. Health-related stories from peers are particularly valued among adolescents [7,13]. Influencers on social media, adolescents who create content, and microcelebrities are increasingly important resources for health-related information and social support [7,14-16]. However, adolescents may find the sheer volume of online information challenging and are not always confident in their ability to discern accurate information from misinformation or disinformation [7].

Social media platforms that are based on the creation of visual media, such as TikTok, are popular with adolescents. TikTok, a video-based social media platform, has been leveraged as a way to disseminate health-related information, and this has been especially visible during the COVID-19 pandemic [17]. The vast reach of TikTok worldwide offers a unique approach for disseminating information to the millions of users ranging from children to adults. As a social media platform, TikTok creators combine music and dance attached to personal messages that are widely disseminated [17].

Several studies have been conducted on the health-related content on TikTok. These studies indicate that a wide range of issues have been examined, including health promoting and compromising issues. Examples include, but are not limited to, studies related to COVID-19 mitigation, eating disorders, vaping, climate change, and equity issues [12,18-26].

Specific to mental health information and social support, researchers determined that youth and young adults appreciated shared experiences within online discussion forums, citing accessibility, anonymity, inclusivity, sense of control, and mitigation of stigma as valued resource characteristics [12]. Preference for seeking health information online or through peer-to-peer sharing can reflect individuals’ concerns or experiences with nonaffirming or discriminatory health care providers [12]. Furthermore, peer-to-peer health information sharing may fill the gaps in social support from health systems, health-specific information, and insight into “how to live” with chronic diseases including mental health. In particular, select TikTok videos may serve as relevant educational resources for health care professionals’ education and training [12]. However, research exploring mental health content on TikTok is essentially absent from published research literature.

Online social media networks provide valuable opportunities to connect with each other to share experiences and strategies for health and wellness, such as meditation, mindfulness, stress relief, and those specific to mental health conditions [13]. Mental health issues are highly prevalent in adolescents. According to the World Health Organization, “globally, one in seven 10-19-year-olds experiences a mental disorder, accounting for 13% of the global burden of disease in this age group” [27]. Given these statistics, combined with the popularity of TikTok use in this age group [28], the aim of this study was to describe the content of the hashtag #mentalhealth on TikTok.

## Methods

### Data Collection and Analysis

This cross-sectional, descriptive content analysis study included videos with the hashtag #mentalhealth on TikTok. The methods were based on prior research and established methodology [24,29,30]. By using the “discover” function on the TikTok platform and a hashtag search of #mentalhealth, a sample of the first 100 videos was collected. At the time of the study, the hashtag had 25.3 billion views. This was the most viewed hashtag in this area at the time of the study (January 2022). Only English-language videos were considered for this sample. For each video, the date of posting and the number of views, comments, and likes were documented. All videos that included the hashtag #mentalhealth were analyzed and coded for the presence of additional content categories. The content categories included general mental health (nonspecific disorder), anxiety or fear, depression, stress, suicide, self-harm, interpersonal relationships, physical health conditions or variables, child or adolescent mental health, mental health stigma, statistics and the prevalence of mental health disorders or issues, biological and neurological influences of mental health, missing other people or connections due to COVID-19, personal experience, and coping techniques or treatment.

Additionally, the comments associated with each video were viewed and coded inductively for content in the following themes: offering support or validation; mentioning experience with suicide or suicidal ideation; mentioning experience with self-harm; describing an experience with hospitalization for mental health issues; describing other mental health issues or struggles; and sharing coping strategies, experiences of healing, or ways to feel better. All data were collected, categorized, and organized by a single reviewer (CJ), and a random number generator was used to identify a subset of (10%) the videos to be analyzed by a second reviewer (CB) to determine interrater reliability. The interrater reliability score (κ=0.97) indicated a high level of consensus. Microsoft Excel was used to record, organize, and analyze the data collected.

### Ethical Considerations

This study was excluded from institutional ethics board review, as the William Paterson University Institutional Review Board does not review studies that do not involve human participants.

## Results

Overall, the 100 videos studied received 1,354,100,000 views; 266,900,000 likes; and 2,515,954 comments. On average, each video received 13,406,930.69 (SD 8,728,095.52) views; 2,657,425.74 (SD 1,449,920.45) likes; and 24,910.44 (SD 21,035.06) comments. Of the 100 videos, a majority (n=84, 84%) were classified as consumer-generated; only 13 (13%) were classified as influencer- or verified-user–generated, with the remaining classified as heath care professional–generated (n=1, 1%), television- or internet-based news (n=1, 1%), and television-based entertainment (n=1, 1%).

In Table 1, the first column lists the 14 different content categories of the video data and the second column details how many of the 100 videos sampled included this content. The table also includes the number of views, likes, and comments that the videos with these particular features garnered. Relative percentages from the total are included as well. The content category “statistics and prevalence of mental health disorders or issues” was omitted from the table since it was not featured in any of the sampled videos.

The only content category observed in a majority (51/100, 51%) of the videos sampled was “general mental health.” The remaining content categories appeared in less than 50% of the sample. “Personal experience” was the next most prevalent category observed in the videos and it appeared in 40% (40/100) of the sample. The remaining content categories appeared in less than 20% of the sample, with the following 5 content categories appearing in less than 5% of the sample: anxiety or fear, physical health conditions or variables, stress, mental health stigma, and missing other people or connections due to COVID-19.

Table 2 provides information about the themes noted in the videos’ comments. This table shows 6 different themes, the number of videos with comments that reflected these themes, and the associated number of views, likes, and comments of these videos. The most common themes observed in the videos’ comments sections were “offering support or validation” (61/100, 61%) and “describing other mental health issues or struggles” (49/100, 49%).

In total, 32% (32/100) of the videos sampled received more than the overall average number of likes (ie, more than 2.67 million likes). Among these 32 videos, 23 (72%) included comments offering support or validation and 20 (62%) included comments describing other mental health issues or struggles. The remaining themes were included in ≥10 videos: shared coping strategies, experiences of healing, or ways to feel better (10/32, 31%); describing an experience with hospitalization for mental health issues (7/32, 22%); mentioning experiences with suicide or suicide ideation (6/32, 19%); and mentioning experience with self-harm (2/32, 6%).

The frequently used words excerpted from the comments section of the TikTok platform are shown in Multimedia Appendices 1-2. Multimedia Appendix 1 shows the negative sentiments portrayed in the comments that reflect serious mental health concerns, which were expressed in response to participant-posted TikTok videos depicting issues of depression, grief, sadness, anger, loneliness, and trauma. In contrast, Multimedia Appendix 2 shows social support and collective optimism in the comments about the posted TikTok videos. These comments were caring and reflected encouragement, praise, and acceptance. It is important to note that these comments were generally presented in the context of support from those who allegedly have shared experiences or trauma.

**Table 1:** Observed content, views, likes, and comments of 100 TikTok videos on mental health.

Content categories	Videos (N=100), n (%)	Views (N=1,354,100,000), n (%)	Likes (N=266,900,000), n (%)	Comments (N=2,515,954), n (%)
General mental health	51 (51)	703,700,000 (51.97)	149,000,000 (55.83)	1,331,622 (52.93)
Personal experience	40 (40)	638,900,000 (47.18)	128,400,000 (48.11)	1,093,179 (43.45)
Interpersonal relationships	18 (18)	366,200,000 (27.04)	75,100,000 (28.14)	440,502 (17.51)
Depression	13 (13)	213,700,000 (15.78)	39,000,000 (14.61)	294,830 (11.72)
Suicide	13 (13)	151,600,000 (11.20)	31,000,000 (11.61)	414,316 (16.47)
Coping techniques or treatment	9 (9)	53,700,000 (3.97)	12,400,000 (4.65)	144,074 (5.73)
Child or adolescent mental health	8 (8)	142,700,000 (10.54)	28,700,000 (10.75)	200,305 (7.96)
Biological and neurological influences of mental health	8 (8)	91,600,000 (6.76)	15,700,000 (5.88)	238,355 (9.47)
Self-harm	5 (5)	62,600,000 (4.62)	15,000,000 (5.62)	181,375 (7.21)
Anxiety or fear	4 (4)	58,600,000 (4.33)	10,600,000 (3.97)	42,000 (1.67)
Physical health conditions or variables	4 (4)	100,300,000 (7.41)	15,900,000 (5.96)	184,800 (7.35)
Stress	3 (3)	50,900,000 (3.76)	8,300,000 (3.11)	25,100 (1.00)
Mental health stigma	1 (1)	11,500,000 (0.85)	2,200,000 (0.82)	6,405 (0.25)
Missing other people or connections due to COVID-19	1 (1)	49,200,000 (3.63)	10,200,000 (3.82)	68,300 (2.71)

**Table 2:** Themes noted in the videos’ comments and the associated number of views, likes, and comments.

Theme	Videos (N=100), n (%)	Views (N=1,354,100,000), n (%)	Likes (N=266,900,000), n (%)	Comments (N=2,515,954), n (%)
Offering support or validation	61 (61)	884,300,000 (65.31)	178,500,000 (66.88)	1,663,433 (66.12)
Describing other mental health issues or struggles	49 (49)	677,500,000 (50.03)	145,300,000 (54.44)	1,419,131 (56.41)
Sharing coping strategies, experiences of healing, or ways to feel better	16 (16)	301,500,000 (22.27)	64,200,000 (24.05)	448,510 (17.83)
Mentioning experience with suicide or suicidal ideation	14 (14)	171,400,000 (12.66)	37,700,000 (14.13)	369,724 (14.7)
Describing an experience with hospitalization for mental health issues	11 (11)	210,000,000 (15.51)	41,500,000 (15.55)	262,565 (10.44)
Mentioning experience with self-harm	7 (7)	87,000,000 (6.42)	17,100,000 (6.41)	187,339 (7.45)

## Discussion

### Principal Findings

It is important to note the reach of the videos included in this study. With over 1 billion cumulative views, almost half of the assessed TikTok videos included in this study reported or expressed symptoms of mental distress. Other studies have observed the expression of mental ill-health within online social media platforms [31] and expressed concern about potentially traumatic and “triggering” TikTok videos as being detrimental to some viewers [32]. This is especially concerning given the frequency of screen time among adolescents. Many adolescents are predominant TikTok participants, and there is a potential for contribution to poor mental health outcomes from repetitious and prolonged viewing, especially if traumatic events are featured in the videos [32]. Recently, TikTok has acknowledged the substantial impact of their platform on users’ mental health and have provided additional resources in support of user safety, health, and mental wellness [33]. Specific to the issues of mental health, TikTok has produced well-being guides in partnership with the several international mental health organizations to support and uptake optimal messaging on the TikTok platform [34]. However, there is limited insight into the impact of traumatic or “triggering” events posted within the online platform [35], the use and impact of the TikTok mental health resources, or conversely, the health enhancing impact of positive and supportive messaging among youth who engage with social media [36,37]

Over 60% of the videos in this study had associated comments that were supportive and validating; this can signify the importance of the TikTok platform and the hashtag #mentalhealth as a possible “just-in-time” source of social support and personal validation that is made available without the need for planning, scheduling, and financial renumeration. Notably, comments that depict coping strategies were not overly common; they were apparent in about 10% of the videos with high numbers of collective views. Given the seriousness of the topics, these findings point to the fact that videos alone only tell part of the story. Health care professionals are active on social media and, specifically, TikTok [17,38]. Comp et al [17] noted that *TikTok therapists,* some with millions of followers, are actively providing corrective information to mental health misinformation and combatting mental health stigma on TikTok.

However, it is important to note that the videos in this sample were largely posted by consumers and not by health care professionals. Hence, further research is needed on the extent to which corrective mental health information is prevalent on popular mental health hashtags. Further, our findings indicate that health care professionals, particularly those who aim to provide corrective information, should be aware of and attend to both the video content and its related commentary.

### Future Implications

Future research should focus on the potential role of intervention by health care professionals on social media. This would create changes for clinician practice, including raising issues related to the ethics of following patients online, concerns related to fake accounts, the user performance factor assumed as part of the TikTok platform, and mental health assessment of social media consumption [31]. As partnerships between health care professionals and social media platforms emerge, evaluation on effectiveness and best practices will be essential. Interestingly, the call for research related to greater understanding of TikTok health content creators, the integrity of the content, and user reaction and uptake to promote evidence-based information [35] to TikTok users may be contrary to the current appeal and need for accessible health-related resources that are different from mainstream medicine [12].

### Limitations

The findings of this study offer insight into the use of TikTok to discuss mental health issues. However, this study is limited by the cross-sectional design. Further, the inclusion of English-only TikTok posts is limiting; videos posted in other languages may contribute to a more comprehensive understanding of the mental health conversations posted using the hashtag #mentalhealth. Other hashtags that specify a mental health illness or experience (suicide, anxiety, or depression) may have captured different videos than those captured by the generic use of hashtag #mentalhealth. Nonetheless, this study can serve as a foundation for further research to assess both the video content and its associated discussions, which are both important components to consider in studying mental health content on TikTok.

## Appendix

Multimedia Appendix 1 Negative mental health sentiments portrayed in the comments associated with 100 TikTok videos.

Multimedia Appendix 2 Sentiments of social support and collective optimism portrayed in the comments associated with 100 TikTok videos.
