# Supplementary figures and images for: Deconstructing TikTok Videos on Mental Health: Cross-sectional, Descriptive Content Analysis
Source: JMIR Form Res. 2022 May 19;6(5):e38340. doi: 10.2196/38340 (PMC9164092; doi:10.2196/38340)

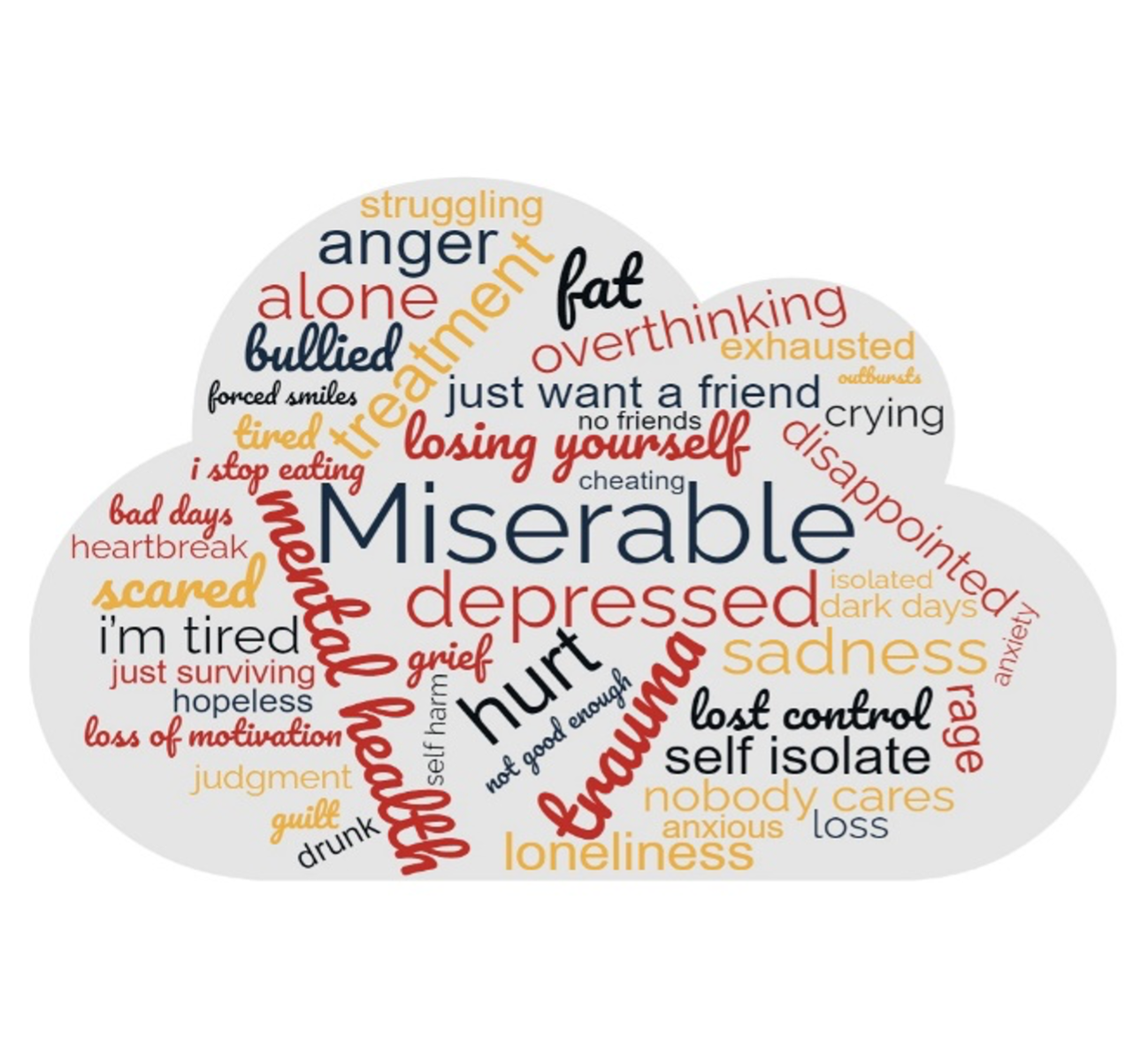

Supplement: Multimedia Appendix 1 [file formative_v6i5e38340_app1.png]

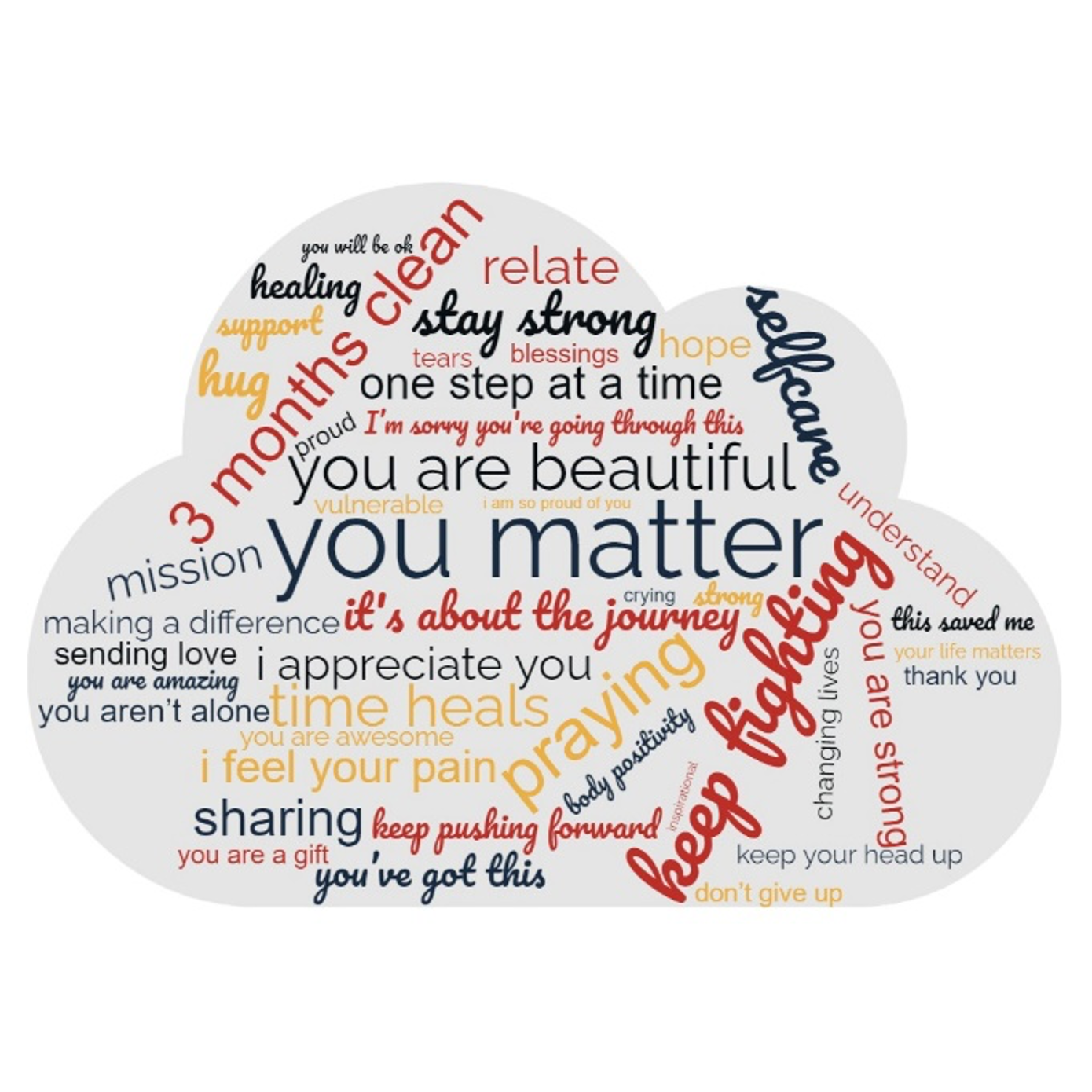

Supplement: Multimedia Appendix 2 [file formative_v6i5e38340_app2.png]
